# Extracellular Vesicles (EVs) Are Copurified with Feline Calicivirus, yet EV-Enriched Fractions Remain Infectious

**DOI:** 10.1128/spectrum.01211-22

**Published:** 2022-07-25

**Authors:** Rachel R. Mizenko, Terza Brostoff, Kenneth Jackson, Patricia A. Pesavento, Randy P. Carney

**Affiliations:** a Department of Biomedical Engineering, University of California, Davis, California, USA; b Department of Pathology, University of California, San Diego, California, USA; c Department of Pathology, Microbiology, and Immunology, School of Veterinary Medicine, University of California, Davis, California, USA; University of Prince Edward Island

**Keywords:** density gradient, extracellular vesicles, Feline calicivirus

## Abstract

Feline calicivirus (FCV) is a major cause of upper respiratory disease in cats and is often used as a model for human norovirus, making it of great veterinary and human medical importance. However, questions remain regarding the route of entry of FCV *in vivo*. Increasing work has shown that extracellular vesicles (EVs) can be active in viral infectivity, yet there is no work examining the role of EVs in FCV infection. Here, we begin to address this knowledge gap by characterizing EVs produced by a feline mammary epithelial cell line (FMEC). We have confirmed that EVs are produced by infected and mock-infected FMECs and that both virions and EVs are coisolated with standard methods of virus purification. We also show that they can be enriched differentially by continuous iodixanol density gradient. EVs were enriched at a density of 1.10 g/mL confirmed by tetraspanin expression, size profile, and transmission electron microscopy (TEM). Maximum enrichment of FCV at a density of 1.18 g/mL was confirmed by titration, quantitative reverse transcriptase PCR (q-RT PCR), and TEM. However, infectious virus was recovered from nearly all samples. When used to infect *in vitro* epithelium, both EV-rich and virus-rich fractions had the same levels of infectiousness as determined by percentage of wells infected or titer achieved postinfection. These findings highlight the importance of coisolates during viral purification, showing that EVs may represent a parallel route of entry that has previously been overlooked. Additional experiments are necessary to explore the role of EVs in FCV infection.

**IMPORTANCE** Feline calicivirus (FCV) is a common cause of upper respiratory infection in cats. Both healthy and infected cells produce small particles called extracellular vesicles (EVs), which are nanoparticles that act as messengers between cells and can be hijacked during viral infection. Historically, the role of EVs in viral infection has been overlooked, and subsequently no group has studied the role of EVs in FCV infection. We hypothesized that EVs may play a role in FCV infection. Here, we show that EVs are copurified with FCV when collecting virus. To study their individual effects, we successfully enrich for viral particles and EVs separately by taking advantage of their different densities. Our initial studies show that EV-enriched versus virus-enriched fractions are equally able to infect cells in culture. These findings highlight the need to both consider the purity of virus after purification and to further study EVs’ role in natural FCV infection.

## INTRODUCTION

Feline calicivirus (FCV) is a member of the *Caliciviridae* family, a family of small (30–40 nm) nonenveloped viruses. Caliciviruses have a single-stranded positive-sense RNA genome of approximately 7.4 to 7.7 kb and contain several important human (norovirus) and veterinary (rabbit hemorrhagic disease virus and FCV) pathogens (1).

FCV was first discovered as an enteric virus but is recognized more widely as a common cause of upper respiratory disease in cats (2–4). FCV infection results in viremia, and thereafter the clinically impactful cellular targets are epithelial, which is a cellular tropism that results in respiratory and/or intestinal disease (5–7). Infection includes penetration of a mucosal layer, establishment of viremia, and basilar introduction to targeted epithelium, but little is known about most of these steps in viral pathogenesis (5). Junctional adhesion molecule-A (JAM-A) is a tight junction protein that is a receptor for FCV which has been identified during *in vitro* infection studies of model cells (8). However, JAM-A is paradoxically expressed buried within the apical junctional complex which exists exactly to prevent epithelial penetration in target cells (9). There are examples in the literature of other viruses modulating epithelial permeability to gain access to the tight junction with their own viral proteins (10, 11). However, a mechanism has yet to be described for JAM-A access in FCV infection. Currently no other route of cellular entry for FCV has been identified.

Extracellular vesicles (EVs) have been identified as potential carriers or facilitators of viral infection (12). EVs are membrane-bound nanoparticles, with a nominal diameter between 30 and 150 nm, which are secreted by cells and can act as a means of intercellular communication (13). EVs may be loaded with functional cargo, such as proteins, receptors, viruses, or small RNAs, in turn with varying effect on target cells when internalized (14–18). EVs are active in healthy tissue function, but they can also be hijacked in many diseases, including cancer (19), neurodegenerative diseases (20), and in viral infection (12). EVs have been found to be associated with a multitude of viruses including human immunodeficiency virus (HIV), Epstein-Barr virus, Hepatitis C virus (12), and human norovirus (21). EVs have been shown to package nonenveloped viruses (17, 22, 23), traffic accessory proteins such as HIV Nef (24), nucleic acids which increase viral replication (25), and even viral receptors to cells which otherwise could not be infected (26). These studies illustrate the immense functional heterogeneity of EVs in viral pathogenesis. Currently, no dedicated study has examined EV association with and function in FCV infection. However, there is precedent for enhanced infectivity of human norovirus in association with EVs in the literature (21).

Viruses are commonly cultured in conditioned media and enriched from cell debris by filtration, noncontinuous density gradient ultracentrifugation, and/or differential ultracentrifugation. During implementation of any of these commonly used isolation methods, EVs would remain intact and could separate with the viral fraction. Specifically, EVs with a nominal density of ~1.1 to 1.2 mg/mL (27–29) would remain in suspension after the ~1,500 × *g* centrifugation that is commonly used to prepare “purified” viral fractions for *in vitro* experiments (30). The same would be true that both EVs and caliciviruses would pass through a 0.22-μm filter. Therefore, it is likely that viruses and EVs are coisolated during routine viral purification from *in vitro* infections. We hypothesize that FCV infection results in the formation of EVs that are coisolated with purified FCV and could play a role in infection of epithelial monolayers. EVs are potential modulators of viral infectivity both *in vitro* and *in vivo*, and if EVs are present in purified viral fractions *in vitro*, it is possible that they contribute to or edify our cell models of viral pathogenesis. Here we characterize and differentially enrich for EVs in purified FCV cultures and examine their potential contribution to the infection of epithelial monolayers.

## RESULTS

For *in vitro* infection studies, FCV is typically isolated from conditioned media by centrifugation at a speed appropriate to pellet higher-density cell debris (~500 × *g*). The resulting supernatant is often either passed through a 0.22-μm filter or simply aliquoted and frozen with no further purification before use in further studies (31–34). Due to their similar size and density, we hypothesized that EVs would remain in the supernatant along with FCV, possibly confounding mechanistic studies.

**FIG 1:**
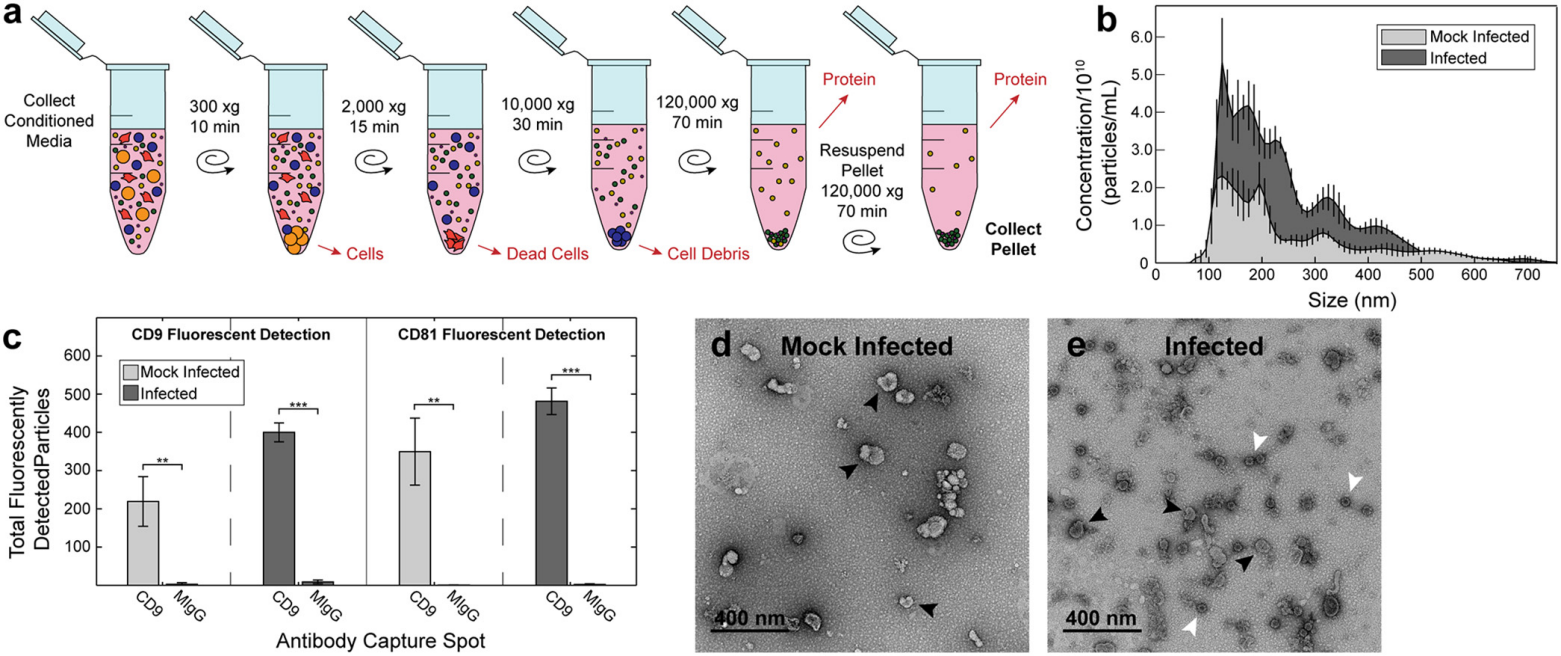
FCV and EVs are both present in samples used for *in vitro* infection. Particles from conditioned media from FMEC culture after mock infection and infection were isolated by differential ultracentrifugation (a). The pelleted particles were then interrogated for size and concentration by NTA (b), tetraspanin content by immunocapture/immunofluorescence (EVs are captured by an immobilized antibody and then labeled using a fluorescent antibody) (c), and morphology by TEM (d and e). Black arrows indicate EVs, and white arrows indicate virions. In panel (c), statistical significance was determined by unpaired *t* test; *n* = 3 technical replicates; *, *P* < 0.05; **, *P* < 0.01; ***, *P* < 0.001.

To examine this, we utilized differential ultracentrifugation (UC), a common method used to enrich EVs according to their sedimentation coefficient (Fig. 1A). Since EVs, with a nominal size of ~30 to 150 nm and density of ~1.1 to1.2 g/mL, have a similar size and density to FCV virions, with a diameter of 40 nm and density of 1.22 to 1.33 g/mL (35), both types of particles should be present in the supernatant after the 10,000 × *g* spin and isolated after the 120,000 × *g* spin using UC.

We collected the final pellet following UC of conditioned media from FMEC monolayers that were either mock infected or infected by FCV. Nanoparticle tracking analysis (NTA), a light scattering based method that can detect the upper size range of EVs (>~90 nm in diameter), was used to confirm that large structures (>200 nm) were depleted during isolations. Particles from both mock-infected and infected samples had the expected size histogram with exponential increase in particle number with decreasing size to the limit of detection of the instrument (Fig. 1B). To confirm that the isolate contained EVs, the presence of tetraspanins (CD9 and CD81), which are EV-associated proteins, was tested by immunocapture/immunofluorescence. The tetraspanin profile of each UC-isolated fraction was similar, suggesting that EVs are produced by both mock and infected cells (Fig. 1C). The presence of both EVs and virions was confirmed by negative stain transmission electron microscopy (TEM). EVs had the typical deflated cup shape (36, 37), an artifact of the drying process, and represented the full size range of EVs of ~30 to 150 nm (Fig. 1D and E). Uniform 40 nm particles with dense cores appeared in the infected sample similar to FCV virions seen previously by TEM (38) (Fig. 1E) and were not present in the mock-infected sample (Fig. 1D). To confirm that particles in the isolate were infectious, viral titer of the infected sample was determined by TCID_50_. While the mock-infected sample had no detectable infectious material, the infected sample had a viral titer of 4.31 × 10^9^ TCID_50_/mL and was not affected by overnight refrigeration at 4°C or freeze at −80°C. Together this confirmed that both virions and EVs are present in the conditioned media harvested by the method historically used for *in vitro* infections.

**FIG 2:**
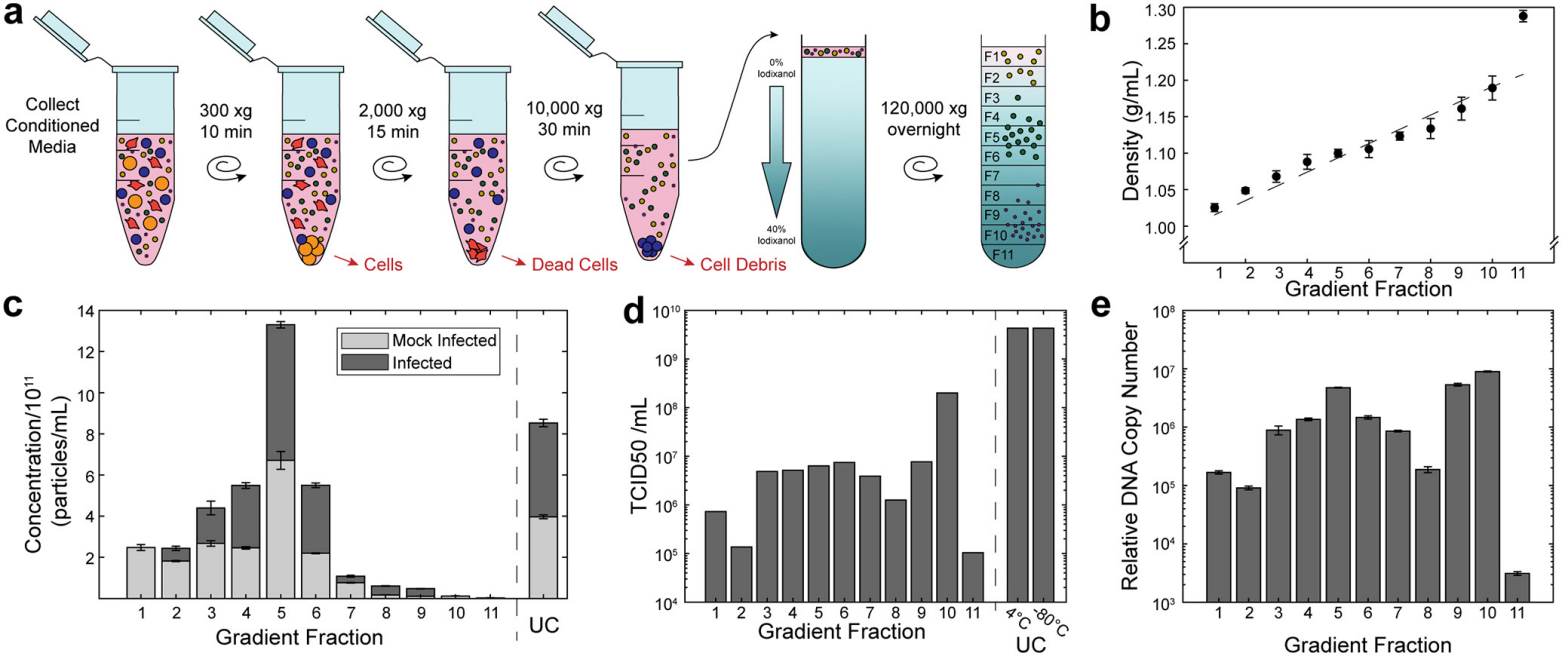
FCV and EVs are enriched at different densities within iodixanol gradient. Conditioned media was depleted of cells, dead cells, and cell debris, prior to subjecting it to ultracentrifugation on an iodixanol gradient and fractionation (a). Fractions from experimentally prepared density gradients (dot) was close to the theoretically perfectly linear density gradient (dashed line) (b). The fractions of mock-infected and infected samples were interrogated for EV presence by NTA (c) and for virus by TCID_50_ (d) and qRT-PCR (e). For panel (c) and (d), statistical significance was determined by ANOVA followed by Tukey’s honestly significant difference; *n* = 3 technical replicates.

To study the differences in these populations we employed an iodixanol density gradient, which has previously been used to separate EVs from viral particles in conditioned media based on their difference in density (39). However, no studies to date have attempted separation of FCV from EVs or characterized a resulting EV profile. Since EVs have a wide range of reported densities, our first goal was to determine if FMEC-derived EVs had a large enough density difference from FCV virions to be enriched in separable fractions of the density gradient. Therefore, we subjected mock-infected and infected conditioned media to iodixanol density gradient ultracentrifugation (Fig. 2A). We produced a continuous gradient using the Gradient Master on the BioComp Gradient Station ranging from 0% to 40% iodixanol. By weighing known volumes from each fraction of three separate density gradients, we experimentally confirmed preparation of a linear gradient from 1.01 to 1.21 g/mL with only slight deviations from a very dense final fraction (Fig. 2B). Concentrated conditioned media was added to the prepared gradients and ultracentrifuged overnight to allow the particles to settle at their isopycnic densities. The gradient was then fractionated by the Piston Gradient Fractionator on the BioComp Gradient station.

The presence of EVs in each fraction was determined by analyzing nanoparticle concentration. By NTA, which is insensitive to viruses but can detect the larger sizes of EVs, there was a spike in concentration of nanoparticles in fraction 5 (F5) with some particles appearing in lower density fractions 1 to 4 but very few remaining in high density fractions 6 to 11 (Fig. 2C). This spike in concentration in F5 was statistically significant compared to all other fractions for both infected (*P* < 0.001) and mock-infected samples (*P* < 0.001). This corresponded to an average density of 1.10 g/mL, which is within the range reported for EVs (40). This suggested that the FMEC EVs were mainly found at F5, a much lower density than that reported for FCV (35). Furthermore, both gradients of mock-infected and infected samples had similar concentration histograms, indicating that the density of detectable EVs was not modified by the presence of virus.

To assess the location of infectious particles in the gradient, TCID_50_ was performed on each sample. For the infected samples, relatively low titers were identified for fractions 1, 2, 8, and 11 (< 1 × 10^6^ TCID_50_/mL) with slightly higher titers associated with fractions 3 to 6 and 9 (1 × 10^6^ – 1 × 10^7^ TCID_50_/mL) (Fig. 2D). However, fraction 10 (F10) contained the most infectious particles with a titer at least 25× higher than any other fraction (2 × 10^8^ TCID_50_/mL). In comparison, the mock-infected sample showed no signs of cytopathic effect (CPE) at these dilutions in any fraction. This suggested that the FCV infectious virus was enriched in F10 at 1.18 g/mL, which was slightly lower than the reported expected density for FCV (35).

The presence of infectious particles in many of the lower density fractions suggested that some infectious material could also be associated with EVs. Since EVs have been shown to carry accessory nucleic acids for other viruses, we hypothesized that some of the CPE could be due to viral RNA carried by EVs. q-RT PCR of FCV RNA revealed that while F10 had the highest relative cDNA copy number (*P* < 0.001), fraction 9 had the second highest (*P* < 0.001), and fraction 5 had the third highest (*P* < 0.001) compared to less concentrated fractions, with fractions 3, 4, 6, and 7 having 10-fold less (Fig. 2E). Fractions 1, 2, 8, and 11 were significantly (50-fold) less concentrated than F10 (*P* < 0.001). C_t_ for the mock-infected sample was above the detectable limit, suggesting that no viral RNA was in this sample (data not shown).

**FIG 3:**
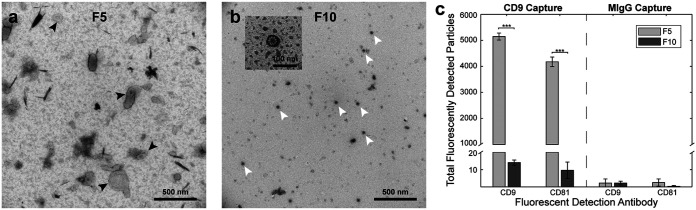
EVs are enriched in F5, and virions are enriched in F10. Negative stain TEM showed classic EVs (black arrows) in F5 (a) and dense core virions (white arrows) in F10 (b). Tetraspanin content was only detected in F5, further confirming that EVs were not present in F10 (c). In panel (c) statistical significance was determined by unpaired *t* test; *n* = 3 technical replicates; *, *P* < 0.05; **, *P* < 0.01; ***, *P* < 0.001.

To further confirm that F5 and F10 were the fractions of enrichment for EVs and FCV virions, respectively, the fractions were imaged by TEM. Although F5 appeared to have a large amount of background, EVs could be identified within the expected size range and with the typical morphology (Fig. 3A) (36, 37). Furthermore, this fraction was devoid of the much smaller dense core FCV particles. In contrast, F10 had a high concentration of FCV particles and was devoid of larger particles (Fig. 3B). Furthermore, relatively high presence of CD9 and CD81 in F5 and nearly undetectable quantities in F10 supported that EVs pooled in F5 (Fig. 3C).

While infectious virus was enriched in fraction 10, as determined by TCID_50_ and corroborated by q-RT PCR, there was quantifiable infectious material present in fraction 5 as well. Because fractions were enriched differentially for EVs, we sought to determine if there is an *in vitro* difference in viral titer achieved by these fractions during the anticipated exponential growth phase. We compared this measurement using both FMEC and CRFK cells, as FMEC (epithelial) cells may provide a more natural model of infection *in vitro*. A growth curve was determined with 500 TCID_50_/well on a 24-well subconfluent plate of each cell type and a time point of 8 hpi was selected based on the growth of stock virus (Table S1). Despite stock virus growing predictably in each well, the density gradient fractions were less efficient at infection for each cell line at this time point, using 10 replicates per condition tested (Table 1). For each fraction on each cell line, the range of viral titers produced were similar and spanned approximately 2 logs. This wide range of growth and mixed productive and nonproductive infection was not observed for the stock virus (Table S1). In summary, F5 and F10 alone are each less efficient at establishing infection at the same multiplicity of infection (MOI) *in vitro* compared to virus isolated by classical methods, which contains a mixture of EVs and viral particles.

**TABLE 1:** Characteristics of FCV infection with (F5) and without (F10) EV enrichment

Cell type	% of 10 wells infected	Titer range (TCID_50_\mL)
CRFK		
Fraction 5	60%	5.37*10^2^–3.17*10^4^
Fraction 10	40%	2.62*10^2^–4.01*10^4^
FMEC		
Fraction 5	70%	3.17*10^2^–7.46*10^4^
Fraction 10	70%	1.12*10^3^–1.59*10^4^

## DISCUSSION

There is mounting evidence that EVs are active in infection of many viruses, with a wide range of functions and impact on pathogenesis. With this budding understanding of the activity of EVs in viral infection, there are two main questions that arise: (i) what implications do EV-associated infectivity have on previous work, and (ii) are EVs an important player in infection of all viruses? The answers to these questions will vary for individual viruses, but conclusions may reiterate universal themes. In this study, we examine these questions for the first time in the context of FCV and show that EVs are coisolated with viruses and that EV-enriched fractions contain infectious material. Each have implications for the fields of virology and EV biology.

We confirm by multiple standard EV characterization techniques that traditional viral purification methods do in fact coisolate EVs with FCV. This indicates that traditional *in vitro* or *in vivo* viral entry studies inadvertently include both virions and the bioactive, potentially entry-modulating EVs. This unintentional coisolation is likely not limited to FCV alone. Many other biological particles have similar physical properties that leads to their coisolation with EVs (41, 42). In this case, FCV is coisolated due to a similar size and density to EVs. Moreover, many viruses can overlap with these characteristics of EVs (43). This suggests that mechanistic studies of some viruses may be confounded by the previously unknown presence of EVs.

Detangling the contribution of the virus itself from coisolated EVs produced during infection remains challenging. Deciphering these separate contributions of EVs and viruses to disease is limited by this general lack of awareness of the presence of EVs in viral preparations, few appropriate technologies to measure EVs and viruses across their similarly nominal sizes, and an inability to separate them. Biologists have long considered defective viral particles and their potential contribution to virus-host interactions, which also share many similarities with EVs (44, 45). However, given the relatively young field of EV biology, such studies have yet to be performed specifically for the contribution of EVs to many viral infections.

To this end, we have enriched EVs separately from FCV by density gradient, taking advantage of the higher density of caliciviruses than the majority of EVs. We clearly demonstrate an enrichment of virions and EVs in separate fractions by NTA, viral infectivity, q-RT PCR, immunocapture, and EM. This method could be used to differentially enrich and characterize multiple viruses and associated EVs.

Infectious FCV material, though enriched in F10 with confirmed virions, was identified in multiple fractions, indicating that the biological state and properties of this infectious material was not uniform. This is consistent with the diverse ways that EVs have been shown to contribute to infectivity of different viruses. Here, both EV-rich and EV-poor density gradient fractions contained viral RNA and were able to initiate infection *in vitro*. However, it is unclear whether naked viral RNA or intact virions were packaged within EVs. We did not visualize EVs directly associating with or enveloping viral particles by TEM. While this lack of a finding can neither confirm nor deny that the two types of particles directly interact, it raises additional questions about how each may modify the others’ activity. It is possible that their direct interactions are disrupted by the processing required for TEM, or that they may interact *in vivo* but not *in vitro*. Equally intriguing is the possibility that they do not interact directly but can provide factors in *trans* and thereby modify their biological activities in an indirect manner.

EVs could contribute in complex ways beyond delivery of RNA to enhance and/or hinder viral infection, and this role is likely best understood in an *in vivo* model with a functioning immune system. While JAM-A has been established as a sufficient entry receptor for FCV, its location within the tight junction renders it inaccessible for intact epithelium *in vivo* (9). While alternative mechanisms of entry have not been proposed, there is precedent for nonenveloped viruses to use EVs for entry (21, 46, 47) or for EVs to deliver necessary receptors for virion entry (26). This is one potential mechanism by which viruses may reach a cell for initial infection, prior to later basolateral cell-to-cell spread. Here, we confirmed that EVs and FCV are coisolated in common virus purification steps and thus could represent a different entry route; however, more work is necessary to understand the mechanism of EV-associated FCV infection.

Additionally, while mammary epithelium is a better model of the natural target of FCV infection than the historical model of CRFKs, additional differences between mammary and oral/respiratory and intestinal mucosal epithelium exist. For this reason, these cell lines may not be an ideal *in vitro* model of infection.

Despite the limitations of *in vitro* infection, continuous cell lines provide a simple and effective baseline to examine interactions between viruses and EVs. These studies are very challenging to perform *in vivo*, and many of the conclusions about the EV/viral interplay have been drawn exclusively from *in vitro* experiments. More studies are required in this area to determine how, or if, EVs enhance or interfere with FCV entry and infection in cats.

## MATERIALS AND METHODS

### Cells and virus culture.

Feline mammary epithelial cells (FMEC) were cultured as previously described (9). Briefly, cells were maintained in Eagle’s minimal essential medium supplemented with heat-inactivated 10% fetal bovine serum (FBS, Genesee, CO, USA), 100 units/mL penicillin, 100 μg/mL streptomycin, 25 ng/mL amphotericin B, 1× nonessential amino acids (GenClone), 1× insulin-transferrin-selenium supplement (Sigma-Aldrich, LA, USA), 10 μg/mL epidermal growth factor (Corning) at 37°C, and 5% CO_2_. All cell culture reagents were purchased from Gibco/Thermo Scientific, MA, USA except as noted. FBS containing media was EV-depleted via ultracentrifugation at 120,000 × *g* at 4°C overnight. Crandell-Rees feline kidney (CRFK; ATCC #CCL-94) cells were grown in minimal essential medium with Earle’s balanced salts, supplemented with 10% FBS, 100 units/mL penicillin, 100 μg/mL streptomycin, 1 mM sodium pyruvate, and 1× nonessential amino acids, and incubated as described above.

A previously described strain of virulent systemic feline calicivirus (FCV-Kaos) was used for *in vitro* infection studies and downstream analysis (48). FMECs were infected at 80% confluence with FCV-Kaos at an MOI of 0.1, and virus was allowed to adsorb for 1 h, then washed 3 times with Earle’s Balanced Salt Solution prior to replacement of EV-depleted maintenance media. Supernatant was harvested at 24 h postinfection and stored at −80°C for downstream assay. Immediately prior to infection (at 80% confluence), cell culture supernatant was harvested as an mock-infected control.

### Differential ultracentrifugation.

EVs and virus from conditioned media were isolated as previously described (49). Briefly, conditioned media was centrifuged at 300 × *g* for 10 min to pellet cells. Supernatant was transferred to a new tube and centrifuged at 2,000 × *g* for 15 min to remove dead cells. Supernatant was transferred to a new tube and centrifuged at 10,000 × *g* for 30 min to remove microvesicles and cell debris. Supernatant was transferred to a new tube and diluted, if necessary, in 0.2 μm filtered PBS and ultracentrifuged at 120,000 × *g* for 70 min to pellet EVs and virions. Free protein containing supernatant was discarded, the pellet was resuspended in filtered PBS and ultracentrifuged at 120,000 × *g* for 70 min. Supernatant was again discarded, the pellet was resuspended in roughly 250 μL of PBS, and frozen in aliquots.

**Iodixanol density gradient.** 12 mL of conditioned media was concentrated using 100 kDa or smaller pore size centrifugal filters (Amicon-MilliporeSigma, MA, USA). Media was spun at 4,000 × *g* for 1 h at 4°C. Concentrated media was diluted to 1 mL using 0.2 μm filtered PBS.

A density gradient of 0 to 40% iodixanol was prepared using Optiprep Density Gradient Medium (SigmaAldrich, WI, USA). Isosmotic stock solution (0.25 M sucrose, 1 mM EDTA, 10 mM TrisHCl) was used for 0% iodixanol solution and dilution of Optiprep for 40% iodixanol solution. A continuous gradient was prepared utilizing the BioComp Gradient Station with provided accessories for the SW40 rotor (Beckman Coulter, IN, USA). Open top ultra-clear tubes were filled halfway using the marker block with 0% iodixanol solution. A long needle was used to fill the tube from the bottom with 40% iodixanol. Care was taken to move the needle upwards while filling to minimize disturbance. The Gradient Master portion of the Gradient Station was utilized for gradient preparation following the provided general protocol. For specifically preparing a continuous 0 to 40% iodixanol gradient for SW40 tubes with long caps, a company-validated protocol was used including the following parameters (time/angle/speed): M#1 3:00/55.0/30, M#2 0:05/84.0/30, M#3 0:10/84.0/0 with series: 1232323232323. Then, 1 mL concentrated conditioned media was carefully added to the top of the gradient and spun overnight at 120,000 × *g* at 4°C.

Gradients were fractionated using the general protocol for the Piston Gradient Fractionator. Specifically, tubes were fractionated at 0.13 mm/s, 8.2 mm per sample, 82 mm tubes, 10 total fractions, producing approximately 1.3 mL/fraction. No wash with sample blow-out was utilized between fractions. Fractions were collected in separate tubes and any remaining iodixanol was resuspended in filtered PBS as fraction 11. To remove gradient forming iodixanol, fractions were added to 100 kDa centrifugal filters (Amicon) and spun at 14,000 × *g* for 5 min. After each fraction had been concentrated, PBS was used for a final wash using the same procedure. Filters were then flipped in new tubes and recovered at 1,000 × *g* for 1 min to collect the concentrated sample. Samples were aliquoted and frozen at −80°C.

### Viral quantification.

For TCID_50_ quantification, serial 1:10 dilutions of samples were incubated with CRFK cells at 80% confluence in 96-well plates in replicates of 8. At 3 to 4 days postinoculation, wells containing cells showing CPE were counted, and TCID_50_/mL were calculated by the Reed and Muench method (limit of detection: 2.62*10^2^ TCID_50_/mL) (50).

### q-RT PCR.

Nucleic acid from density gradient and differential ultracentrifugation samples was isolated using the PureLink Viral RNA/DNA minikit (Thermo Scientific) and cDNA synthesis was performed using the QuantiTect reverse transcription kit (Qiagen, MD, USA) according to manufacturer’s instructions. Real-time PCR was performed on an ABI 7500-fast cycler (Applied Biosystems, MA, USA) using a previously published assay further validated in-house (51). Amplifications were performed using Maxima SYBR green with ROX as a reference dye (Thermo Scientific). All samples were assayed in triplicate, with positive and negative controls included in each run. For quantitative analyses and comparisons, the cycle threshold (Ct) value was used.

### Nanoparticle tracking analysis (NTA).

EVs were diluted in 0.2 μm filtered PBS to between 1 × 10^7^ and 2 × 10^9^ particles/mL and loaded by syringe pump (Harvard Bioscience, MA, USA). The NanoSight LM10 (Malvern Panalytical Ltd., UK) was used for data collection with NanoSight NTA 3.1. software for analysis. Three 90-sec videos were collected to determine an average concentration and size profile of particles with camera level of 12 and detection threshold of 3. Between samples, MilliQ water was used to clear out the sample lines.

### Fluorescence microscopy.

ExoView Tetraspanin kits (NanoView Biosciences, MA, USA) were used to quantify tetraspanins on isolated EVs. The company-provided instructions were followed for chip incubation and imaging. In brief, EVs were diluted in 1× Solution A and incubated on individual chips overnight in 24-well plates. Chips were washed in 1× Solution A in 4 successive steps by adding Solution A to each well to reach a total volume of 1 mL per well, mixing on shaker for 3 min, and removing 750 μL of the solution. The fluorescent antibody solution was prepared as recommended by the company with the following antibody/fluorophore combinations: CF488-anti-CD9 (clone: HI9a), and CF555-anti-CD81 (clone: JS 81). Next, 250 μL of this solution was added to each well and allowed to incubate on the shaker at room temperature for 1 h. Chips were immediately rinsed with 1 mL of Solution A followed by removing 750 μL of the solution from each well. Chips were then washed as described previously with 5 successive steps: once with Solution A, three times with solution B, and one final time with MilliQ water. The chips were then dried by swirling each chip in MilliQ water and dried by lifting chips slowly at a 45-degree angle out of solution.

Chips were imaged using nScan software. Detection thresholds were chosen by limiting the number of detected particles on the mouse immunoglobulin (MIgG) capture spot, representing nonspecific capture, to ~10 particles. The following thresholds were used for the blue and green channels: 600 and 400.

### Transmission electron microscopy (TEM).

EVs or virus were prepared for TEM by methods described previously with slight alterations (52). Isolated particles were mixed 1:1 with 4% paraformaldehyde (Electron Microscopy Sciences, PA, USA). A 5 to 10 μL drop of this mixture was put on parafilm covering a glass slide. A copper Formvar TEM grid (VWR International, CA, USA) was floated on the droplet for 20 min to allow particles to adsorb. The grid was transferred to a PBS droplet to wash away free particles, then to a droplet of 2% glutaraldehyde (Electron Microscopy Sciences, PA, USA) for 5 min. The grid was then washed in 8 successive droplets of MilliQ water. For negative staining, this droplet was then transferred to a droplet of uranyl oxalate for 5 min. The grids were then washed in a droplet of MilliQ water then dried by wicking excess liquid on filter paper. EVs were imaged on a Talos L120C (Thermo Scientific).

### Differential fraction infection study.

Material isolated from density gradient fractions 5 and 10 were separately used to infect both FMEC and CRFK cells. A total of 500 TCID_50_ per well per fraction were used to infect each of 10 wells of a 24-well plate seeded with either FMEC or CRFK cells at 80% confluence. Virus was allowed to adsorb for 1 h, then maintenance media was replaced at time zero. At 8 h postinfection, supernatant was harvested, and viral titer measured by TCID_50_ as described above.

### Statistical analysis.

Statistical analysis of immunocapture/immunofluorescence was completed by unpaired t test, α = 0.05. Statistical analysis of NTA and qPCR data was completed by ANOVA (α = 0.05) and, if *P* < α, was followed by Tukey’s HSD post hoc test (α = 0.05). All error bars in figures represent standard deviation of three replicates.

### Data availability.

All raw data generated and analyzed for this study are available from the Zenodo database at https://zenodo.org/record/5903379#.YtdDZXbMK3A.
